# Anti‐interleukin‐23 for psoriasis in elderly patients: guselkumab, risankizumab and tildrakizumab in real‐world practice

**DOI:** 10.1111/ced.14979

**Published:** 2021-11-17

**Authors:** A. Ruggiero, G. Fabbrocini, E. Cinelli, S. S. Ocampo Garza, E. Camela, M. Megna

**Affiliations:** ^1^ Section of Dermatology Department of Clinical Medicine and Surgery University of Naples Federico II Naples Italy; ^2^ Department of Dermatology University Hospital Dr José Eleuterio González Universidad Autónoma de Nuevo León Monterrey Mexico

## Abstract

**Background:**

Elderly patients (aged ≥ 65 years) represent an increasing proportion of patients with psoriasis and 15% of these have moderate to severe disease. Biologics are being used frequently in this group of patients even though safety and efficacy data are limited. In addition, owing to anti‐interleukin (IL)‐23 therapies being a relatively recent option, no data have been reported about their use in elderly patients with psoriasis.

**Aim:**

To evaluate and compare the safety and efficacy of guselkumab, risankizumab and tildrakizumab in real‐world practice in elderly patients.

**Methods:**

This was a single‐centre retrospective study that enrolled patients aged ≥ 65 years with moderate to severe plaque psoriasis, treated with guselkumab, risankizumab or tildrakizumab. The length of the study for each group depended on the drug (44 weeks for risankisumab, 40 weeks for guselkumab and 28 weeks for tildrakizumab, owing to its more recent availability in Italy).

**Results:**

In total, 34 patients were enrolled (*n* = 20 on guselkumab; *n* = 8 on risankizumab; *n* = 6 on tildrakizumab). At Week 4, 29.4% reached 90% improvement in Psoriasis Area and Severity Index (PASI90) and 8.8% reached 100% improvement in PASI (PASI100); at Week 28, PASI90 and PASI100 was reached by 58.8% and 29.4%, respectively. At the final follow‐up (Week 40 or 44, depending on drug), data were available only for the risankizumab (Week 40) and guselkumab (Week 44) and groups, and showed that 71.4% of patients had reached PASI90 and 53.5% had reached PASI100. Four patients (11.7%) discontinued treatment. No significant differences were found between the three groups. The limitations of the study included its retrospective nature of the study, small sample size, and different numbers of patients and follow‐up duration for the different groups (highest for guselkumab, lowest for tildrakizumab).

**Conclusion:**

The three anti‐IL‐23 therapies assessed are promising, safe and effective options in elderly patients, and there was no significant difference between them. However, more data are needed to confirm our results and to understand their role in the management of this group of patients.

## Introduction

Psoriasis is a chronic inflammatory disease affecting 1%–3% of global population.^1^ Although its prevalence is higher in patients aged between 16–20 and 57–60 years, psoriasis may occur at any age.^2^ Patients aged ≥ 65 years represent an increasing proportion of the psoriasis population, with 15% of elderly patients with psoriasis having moderate to severe disease.^3^ Psoriasis management can often be challenging in this group, owing to the frequency of multiple comorbidities and linked polypharmacy, infections and cancer vulnerability.^4^, ^5^ However, to date, international guidelines or shared recommendations about psoriasis treatment in elderly patients are lacking. A possible option is narrowband ultraviolet B phototherapy, but it requires regular visits and adequate patient mobility, which are often not practical for elderly patients.^4^, ^6^

Conventional systemic treatments including acitretin, methotrexate and ciclosporin, are often avoided due to the risk of adverse events (AEs) and frequent contraindications. Hence, elderly patients with moderate to severe psoriasis may be undertreated, resulting in severe psychological and physical impacts.^4^ Biologics are often used even though safety and efficacy data in the elderly are limited; indeed, patients aged ≥ 65 years are frequently excluded from clinical trials. A recent systematic review supported biologic agents use in elderly patients, although it highlighted that serious AEs and treatment discontinuation due to AEs were more common in older patients.^7^ However, recent real‐life studies have shown biologics to be a safe and effective treatment option, with most studies evaluating anti‐tumour necrosis factor (TNF)‐α, anti‐interleukin (IL)‐17 and anti‐IL‐12/23.^4^, ^8^, ^9^, ^10^, ^11^, ^12^ Anti‐IL‐23 drugs represent the latest class of biologics approved for the treatment of moderate to severe psoriasis. For guselkumab, risankizumab and tildrakizumab, promising results in terms of safety and efficacy have been shown in both clinical trials and real‐life studies.^1^, ^13^, ^14^, ^15^, ^16^, ^17^, ^18^, ^19^, ^20^, ^21^ However, because these are new therapies that have only recently become available, no real‐life data on elderly patients have been reported to date. We report the results of a real‐world practice retrospective study evaluating and comparing the safety and efficacy of guselkumab, risankizumab and tildrakizumab in elderly patients with moderate to severe psoriasis.

## Methods

The study was approved by the ethics committee of the University of Naples Federico II (Naples, Italy), and performed in accordance with the principles of the Declaration of Helsinki. All patients signed an informed consent before starting the study, and gave consent to publication of case details and images, where applicable.

### Study design

This was a single‐centre retrospective study performed in Italy, which enrolled patients with moderate to severe psoriasis who attended the Psoriasis Care Center, University of Naples Federico II, Naples, Italy during the period October 2018 to March 2021.

### Inclusion criteria

Inclusion criteria were: (i) moderate to severe plaque‐psoriasis diagnosis since at least 1 year; (ii) patients aged ≥ 65 years; (iii) patients starting treatment with guselkumab, risankizumab or tildrakizumab; and (iv) treatment period of at least 44 weeks for risankisumab, 40 weeks for guselkumab and 28 weeks for tildrakizumab (owing to its more recent availability in Italy).

### Data collection

At baseline, the following items were registered for each patient: (i) personal and demographic data; (ii) duration of psoriasis and any eventual psoriatic arthritis (PsA); (iii) comorbidities; (iv) previous systemic treatments for psoriasis; and (v) psoriasis severity as measured using the Psoriasis Area and Severity Index (PASI) and body surface area (BSA) tools.

At every follow‐up, psoriasis severity (PASI and BSA), routine blood tests [complete blood count, transaminases, creatinine, azotaemia, glycaemia, erythrocyte sedimentation rate, C‐reactive protein, levels of total cholesterol and triglycerides, protein electrophoresis] and AEs were evaluated. Safety was assessed by treatment‐emergent AEs, physical examination and laboratory monitoring.

### Statistical analysis

Data are presented as mean ± SD (for continuous variables) or as number and percentage of patients (for categorical variables). The significance of differences in mean values obtained at the different time points of treatment was assessed by the unpaired Student *t*‐test, with *P* < 0.05 considered statistically significant. All statistical analyses were performed using GraphPad Prism software (version 4.0; GraphPad Software Inc., La Jolla, CA, USA).

## Results

### Patients

In total, 34 patients were enrolled [19 men (55.9%), 15 women (44.1%); mean ± SD age 68.9 ± 5.1 years]. Psoriasis duration was 22.6 ± 14.1 years. Half of the patients (52.9%) also had PsA. The most frequent comorbidity was hypertension (70.5%), followed by dyslipidaemia (47.1%), diabetes (32.3%) and depression (26.4%). All patients had been treated with at least one conventional systemic drug, with methotrexate (67.6%) and ciclosporin (35.3%) being the most common.

### Previous biologic treatment

Only 8.8% of the patient group had never received biologics, while 91.2% had been treated previously with at least one biologic, of whom 85.2% had failed to respond to an anti‐TNF, 35.3% to an anti‐IL‐12/23 and 67.6% to an anti‐IL‐17 drug. Mean anti‐IL‐23 treatment duration was 43.7 ± 8.2 weeks.

### Clinical outcomes

**Figure 1:**
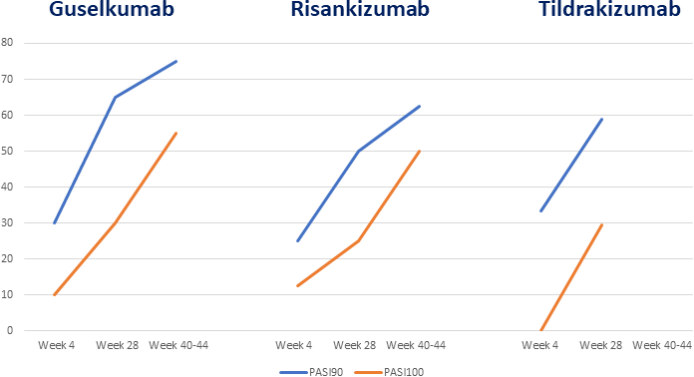
Response rates of 90% and 100% improvement in Psoriasis Area and Severity Index (PASI90 and PASI100 respectively) in guselkumab, risankizumab and tildrakizumab groups during follow‐ups (Week 40/44 follow‐up was not available for tildrakizumab, owing to its more recent availability in Italian routine clinical practice).

The mean clinical outcomes were 90% and 100% improvement in PASI (PASI90 and PASI100 respectively). In the total patient group, 29.4% reached PASI90 and 8.8% PASI100 at Week 4, while at Week 28, PASI90 and PASI 100 were observed in 58.8% and 29.4%, respectively, and at the end of the study (Weeks 40–44, depending on drug), 71.4% had reached PASI90 and 53.5% PASI100. There were no statistically significant differences in efficacy between the three drugs, except for PASI100 at Week 4, which was lower for the tildrakizumab group compared with the other two groups (Fig. 1).

### Discontinuation

Only four patients (11.7%) discontinued the treatment: three patients stopped because of secondary inefficacy; one patient stopped because of a diagnosis of bladder cancer 18 weeks after guselkumab initiation. Mild and nonclinically relevant blood test alterations were experienced by 23.5% of patients.

### Adverse effects

Potential registered AEs were found in 29.4% of patients, including pharyngitis (12.5%), headache (12.5%), influenza‐like illness (8.3%) and diarrhoea (2.9%), none of which required treatment discontinuation. Again, no significant differences in discontinuation rate or safety were observed between the three investigated drugs. No other cases of serious AEs, injection site reaction, *Candida* infection, malignancy or cardiovascular events (CVEs) were reported. Detailed data on overall study population and on the three groups separately are displayed in Table 1.

**Table 1:** Characteristics of the study population and clinical outcomes during guselkumab, risankizumab and tildrakizumab treatments.

Parameter	Guselkumab	Risankizumab	Tildrakizumab	Total
Patients, *n*	20	8	6	34
Sex, M/F; *n* (%)	11/9 (55/45)	5/3 (62.5/37.5)	3/3 (50/50)	19/15 (55.9/44.1)
Age, years^a^	70.5 ± 5.9	68.2 ± 7.5	66.6 ± 1.5	68.9 ± 5.1
Psoriasis duration, years^a^	31.1 ± 12.7	18.5 ± 8.9	7.3 ± 3.3	22.6 ± 14.1
Psoriatic arthritis, *n* (%)	10 (50)	5 (62.5)	3 (50)	18 (52.9)
Comorbidities, *n* (%)				
Hypertension	15 (75)	5 (62.5)	4 (66.6)	24 (70.5)
Diabetes	6 (30)	3 (37.5)	2 (33.3)	11 (32.3)
Cardiopathy	5 (25)	2 (25)	1 (16.6)	8 (23.5)
Dyslipidaemia	11 (55)	2 (25)	3 (50)	16 (47.1)
Depression	6 (30)	1 (12.5)	2 (33.3)	9 (26.4)
Previous cancer	1 (5)	0 (0)	0 (0)	1 (2.9)
Monoclonal gammopathy	1 (5)	0 (0)	0 (0)	1 (2.9)
Gastric ulcer	0 (0)	1 (12.5)	1 (16.6)	2 (5.8)
Glaucoma	0 (0)	0 (0)	1 (16.6)	1 (2.9)
Prostatic hyperplasia	3 (15)	2 (25)	1 (16.6)	6 (17.6)
Latent TB infection	2 (10)	1 (12.5)	0 (0)	3 (8.8)
Other	6 (30)	3 (37.5)	2 (33.3)	11 (32.3)
Previous conventional systemic treatments, *n* (%)				
Ciclosporin	8 (40)	2 (25)	2 (33.3)	12 (35.3)
Methotrexate	14 (70)	5 (62.5)	4 (66.6)	23 (67.6)
Acitretin	4 (20)	2 (25)	1 (16.6)	11 (32.3)
NB‐UVB phototherapy	3 (15)	2 (25)	1 (16.6)	6 (17.6)
Previous biologic treatments, *n* (%)				
Anti‐TNF	18 (90)	7 (87.5)	4 (66.6)	29 (85.2)
Adalimumab	9 (45)	5 (62.5)	2 (33.3)	16 (47)
Etanercept	8 (40)	3 (37.5)	0 (0)	11 (32.3)
Infliximab	4 (20)	1 (12.5)	1 (16.6)	6 (17.6)
Certolizumab	3 (15)	1 (12.5)	0 (0)	4 (11.7)
Golimumab	2 (10)	0 (0)	1 (16.6)	3 (8.8)
Anti‐IL‐12/23	7 (35)	4 (50)	1 (16.6)	12 (35.3)
Ustekinumab	7 (35)	4 (50)	1 (16.6)	12 (35.3)
Anti‐IL‐17	16 (80)	3 (37.5)	4 (66.6)	23 (67.6)
Secukinumab	10 (50)	2 (25)	1 (16.6)	13 (38.2)
Ixekizumab	6 (30)	1 (12.5)	2 (33.3)	9 (26.4)
Brodalumab	0 (0)	0 (0)	1 (16.6)	1 (2.9)
Bio‐naïve patients	2 (10)	0 (0)	1 (16.6)	3 (8.8)
Mean treatment duration, weeks	48.6 ± 5.8	43.2 ± 5.4	29.6 ± 3.5	43.7 ± 8.2
Discontinuation rate	(10) 2	(12.5) 1	(16.6) 1	(11.7) 4
Adverse events				
Pharyngitis	(10) 2	(12.5) 1	(0) 0	(12.5) 3
Influenza‐like illness	(5) 1	(0) 0	(16.6) 1	(8.3) 2
Headache	(5) 1	(12.5) 1	(16.6) 1	(12.5) 3
Diarrhoea	(5) 1	(0) 0	(0) 0	(2.9) 1
Measurements				
Baseline				
Mean PASI	17.1 ± 5.1	13.2 ± 5.2	14.8 ± 9.1	15.1 ± 7.2
Mean BSA	34.1 ± 13.5	28.6 ± 12.8	27.5 ± 18.5	30.1 ± 16.2
Week 4				
Mean PASI	5.5 ± 2.8	5.2 ± 3.4	6.2 ± 5.9	5.6 ± 4.8
Mean BSA	12.7 ± 4.9	12.5 ± 6.5	12.8 ± 9.6	12.6 ± 6.9
PASI90	6 (30)	2 (25)	2 (33.3)	10 (29.4)
PASI100	2 (10)	1 (12.5)	0 (0)	3 (8.8)
Week 28				
Mean PASI	2.1 ± 1.2	2.4 ± 1.9	4.1 ± 5.2	2.8 ± 4.7
Mean BSA	6.5 ± 3.6	7.2 ± 4.1	4.7 ± 4.3	6.1 ± 4.2
PASI90	13 (65)	4 (50)	3 (50)	20 (58.8)
PASI100	6 (30)	2 (25)	2 (33.3)	10 (29.4)
Weeks 40–44^b^				
Mean PASI	0.9 ± 1.1	1.1 ± 1.4	N/A	1.0 ± 1.4
Mean BSA	2.2 ± 1.8	3.3 ± 3.7	N/A	2.7 ± 2.6
PASI90	15 (75)	5 (62.5)	N/A	20 (71.4)
PASI100	11 (55)	4 (50)	N/A	15 (53.5)

BSA, body surface area; NB‐UVB, narrowband ultraviolet B; PASI, Psoriasis Area and Severity Index; TB, tuberculosis; TNF, tumour necrosis factor.

^a^ Mean ± SD.

^b^ 40 and 44 weeks for risankizumab and guselkumab respectively.

## Discussion

With ageing populations worldwide, there is a higher proportion of elderly patients with psoriasis.^7^ However, psoriasis management may be challenging in these patients, as they frequently have multiple comorbidities and related polypharmacy, and increased rates of AEs. Additionally, the development of immunosenescence, which is a progressive functional impairment of the immune system related to ageing, may result in an increased susceptibility to infections and cancers, further complicating the approach to treatment of elderly patients.^22^ Despite the fact that moderate to severe psoriasis is increasingly observed in the elderly population, data regarding the therapeutic management of these patients are limited.^22^ Biologic treatments have revolutionized psoriasis therapy,^23^ but data on their efficacy and safety in elderly patients are limited. Indeed, until relatively recently, these patients were not included in randomized clinical trials (RCTs), resulting in a high rate of undertreated patients in real‐world settings and a lack of shared recommendations or guidelines for this age group.^2^

To date, most of the reported relevant clinical studies on biologics in elderly patients have focused on the efficacy and safety of anti‐TNF‐α drugs, owing to their longer market availability.^24^, ^25^, ^26^, ^27^ A *post hoc* analysis of two phase III RCTs of etanercept found no significant difference in terms of efficacy between elderly and younger patients, but older patients did have a higher risk of AEs.^24^ However, a *post hoc* analysis of adalimumab, the REVEAL trial, found that the drug had lower effectiveness in elderly than in younger patients.^25^ More data are available about the safety profile in elderly patients of certolizumab pegol, a drug used to treat rheumatoid arthritis, which showed a higher frequency of serious infections in elderly than in younger cohorts, although the experience in psoriasis is limited.^26^ However, a retrospective multicentric study including 266 elderly patients treated with anti‐TNF drugs (etanercept, adalimumab, certolizumab and infliximab), showed that the prevalence and frequency of AEs was comparable to that seen in younger patients, implying that age could not be used to restrict treatment choices.^27^ Ustekinumab, an anti‐IL‐12/23 therapy, showed a similar or slightly lower clearance rate in elderly patients, with low rates of discontinuation and AEs.^10^, ^28^, ^29^

Regarding anti‐IL‐17 inhibitors, a recent study including 114 elderly patients showed that these drugs were an effective and safe therapeutic option for patients with psoriasis aged ≥ 65 years, with low rates of only mild AEs; however, the discontinuation rate was 28.9%, mostly related to psoriasis relapses.^30^ A *post hoc* analysis of three phase III secukinumab trials (ERASURE, FIXTURE and CLEAR) showed comparable efficacy profiles between elderly and younger patients; however, the rates of serious AEs and discontinuation were higher in older participants.^31^ For ixekizumab, a retrospective observational study showed optimal efficacy and safety in elderly patients throughout a 1‐year treatment period, confirming the results highlighted in the drug product information, reporting that the response in elderly patients seems to be higher than that in younger patients.^12^, ^32^

Owing to their recent availability, data on anti‐IL‐23 therapies for elderly patients in real‐world clinical practice are still scant. Phase III clinical trials showed that anti‐IL‐23 agents have an acceptable safety profile in elderly patients.^33^ Guselkumab was the subject of four phase III trials (VOYAGE‐1, VOYAGE‐2, ECLIPSE and NAVIGATE), which together enrolled 93 participants aged ≥ 65 years and 4 participants aged ≥ 75 years; the studies reported promising results for efficacy and safety in elderly patients, with no differences from younger patients.^13^, ^14^, ^34^, ^35^, ^36^ For risankizumab, phase III studies (UltIMMa‐1 and UltIMMa‐2) included a total of 243 patients aged ≥ 65 years and 24 aged ≥ 75 years, without showing any overall differences compared with younger participants,^18^ while a recent pooled analysis of two phase III trials (reSURFACE 1 and reSURFACE 2), which included 92 participants aged ≥ 65 years and 17 aged ≥ 75 years, reported a 3‐year safety profile of tildrakizumab comparable with placebo, in terms of rates of major CVEs and serious infection.^21^ Even if data about real‐life‐practice are increasing, particularly for guselkumab and risankizumab, to date, real‐world studies focusing on anti‐IL‐23s in elderly patients are still lacking.^15^, ^16^, ^17^, ^18^, ^19^ Our real‐world study showed promising results in terms of both safety and efficacy for all anti‐IL‐23. Indeed, we reported high rates of both PASI90 and PASI100 responses; particularly, PASI90 and PASI100 were respectively reached by 71.4% and 53.5% of patients at Weeks 40–44 for the pooled guselkumab and risankizumab participants. For tildrakizumab, data for Weeks 40–44 were not available because of its more recent availability on the Italian market; however, the Week 28 data for tildrakizumab showed a comparable PASI90 and PASI100 response (50% and 33.3%) to that for the other anti‐IL‐23 drugs. The discontinuation rate was comparable between the three groups, varying from 10% for guselkumab to 16.6% for tildrakizumab. No significant differences in safety were found between the three treatments, with a total rate of 29.4% for AEs for the combined groups (25% for guselkumab, 25% for risankizumab and 33.3% for tildrakizumab; see Table 1 for group differences). The discontinuation rate (total 8.8%) was also similar. Hence, our results confirm recent real‐life data about guselkumab and risankizumab, showing similar clinical outcomes and AE rates.^13^, ^14^, ^15^, ^16^, ^17^, ^18^, ^19^, ^20^, ^21^, ^37^ For tildrakizumab, studies reporting its use in real‐world practice are still lacking to date; however, our data further confirm its promising results from trials in terms of both clinical outcomes and safety. Hence, our data suggest that anti‐IL‐23 drugs are a safe and effective treatment option in elderly patients without significant differences between them.

The limitations of our study included its retrospective nature, relatively small sample size, unequal follow‐up duration among the three groups, the lower number of tildrakizumab‐treated patients and higher components of guselkumab (owing to the very different date of commercialization in Italy).

## Conclusion

The three anti‐IL‐23 therapies assessed are promising, safe and effective options in elderly patients, and there was no significant difference between them. However, more data, both from dedicated trials and real‐life reports, are needed to confirm our results in order to better understand the role that anti‐IL‐23 therapies could play in the management of this group of patients.
